# Implicit, automatic semantic word categorisation in the left occipito-temporal cortex as revealed by fast periodic visual stimulation

**DOI:** 10.1016/j.neuroimage.2021.118228

**Published:** 2021-05-31

**Authors:** Angelique Volfart, Grace E. Rice, Matthew A. Lambon Ralph, Bruno Rossion

**Affiliations:** a University of Louvain, Psychological Sciences Research Institute, B-1348 Louvain-La-Neuve, Belgium; b Université de Lorraine, CNRS, CRAN, F-54000 Nancy, France; c MRC Cognition and Brain Sciences Unit, University of Cambridge, CB2 7EF Cambridge, United Kingdom; d Université de Lorraine, CHRU-Nancy, Service de Neurologie, F-54000 Nancy, France

**Keywords:** Semantic memory, Conceptual categorisation, Scalp EEG, Electrophysiology

## Abstract

Conceptual knowledge allows the categorisation of items according to their meaning beyond their physical similarities. This ability to respond to different stimuli (e.g., a leek, a cabbage, etc.) based on similar semantic representations (e.g., belonging to the vegetable category) is particularly important for language processing, because word meaning and the stimulus form are unrelated. The neural basis of this core human ability is debated and is complicated by the strong reliance of most neural measures on explicit tasks, involving many non-semantic processes. Here we establish an implicit method, i.e., fast periodic visual stimulation (FPVS) coupled with electroencephalography (EEG), to study neural conceptual categorisation processes with written word stimuli. Fourteen neurotypical participants were presented with different written words belonging to the same semantic category (e.g., different animals) alternating at 4 Hz rate. Words from a different semantic category (e.g., different cities) appeared every 4 stimuli (i.e., at 1 Hz). Following a few minutes of recording, objective electrophysiological responses at 1 Hz, highlighting the human brain’s ability to implicitly categorize stimuli belonging to distinct conceptual categories, were found over the left occipito-temporal region. Topographic differences were observed depending on whether the periodic change involved living items, associated with relatively more ventro-temporal activity as compared to non-living items associated with relatively more dorsal posterior activity. Overall, this study demonstrates the validity and high sensitivity of an implicit frequency-tagged marker of word-based semantic memory abilities.

## 1 Introduction

Conceptual knowledge allows categorisation of items above and beyond their physical, superficial properties, which is crucial for semantic-based generalisations (Lambon Ralph and Patterson, 2008; Lambon Ralph et al., 2010, 2017). For instance, neurotypical human adults have an internal knowledge of what a bird is, being able to accept non-prototypical exemplars (e.g., ostrich) and reject similar looking but non-bird objects (e.g., a duck-shaped watering pot) from this category. Although conceptual categorisation is not specific to humans (e.g., Miller et al., 2002) and does not require language, it is greatly facilitated and expanded by language.

The neural basis of this core ability is still debated and, based on a variety of cognitive neuroscience approaches, various brain regions have been implicated (anterior or posterior temporal cortex, pre-frontal cortex, medial temporal structures such as the hippocampus, etc.; Martin and Chao, 2001; Martin, 2007; Patterson et al., 2007; Quiroga, 2012; Lambon Ralph, 2014; Lambon Ralph et al., 2017). Elucidating this issue is complicated by the strong reliance of most neural measures on explicit tasks (e.g., retrieving or naming concepts), which involve many non-semantic processes (e.g., retrieval and working memory processes, attention, decision-related processes, etc.). To make progress in these debates, a robust marker of implicit (i.e., task-free) conceptual categorisation in the human brain would be strongly desirable. The key aim of the current study was thus to establish an implicit method, i.e., fast periodic visual stimulation (FPVS) coupled with electroencephalography (EEG), to the study of neural conceptual categorisation processes.

The FPVS-EEG approach is based on the early report that a periodically-presented visual stimulus (e.g., a flickering light) generates a periodic brain response exactly at the stimulus frequency (Adrian and Matthews, 1934), providing an objective marker of the stimulus processing in the EEG frequency-domain (Regan, 1966 see Norcia et al., 2015 for review). Over the last decade, this highly sensitive approach, also termed EEG frequency-tagging, has been expanded from low-level vision and its modulation by spatial and selective attention (e.g., Müller et al., 2006) to study face (e.g., Rossion and Boremanse, 2011; Rossion et al., 2015) and visual word recognition (Lochy et al., 2015) and their neural basis (Jonas et al., 2016; Lochy et al., 2018). Most recently, the FPVS-EEG approach has been used to measure semantic categorisation using object pictures (Stothart et al., 2017; Milton et al., 2020). However, picture stimuli of different semantic categories such as living and non-living items are intrinsically associated with distinct objective visual features (i.e., most animals have curved shapes, a limited range of colours, and share common physical features such as legs, while manufactured objects can take many forms and colours and are very different from each other). These physical features often cannot (and should not) be neutralized in an experimental design, and will therefore necessarily contribute to any semantic categorisation response.

Unlike pictures, words allow a full exploration of semantics which is important for at least two significant reasons. First, in most cases, there is an arbitrary mapping between form and meaning in words but not in pictures (Lambon Ralph et al., 1999; Gasser, 2004). Second, many concepts can only be expressed through language (e.g., liberty, faith, honesty, etc.) including some emotion or action words (e.g., hate, nostalgia, to believe, to fulfil, etc.). For these reasons, here we use written word stimuli for the first time in a FPVS-EEG paradigm aimed at implicitly probing automatic semantic/conceptual categorisation.

**Fig. 1:**
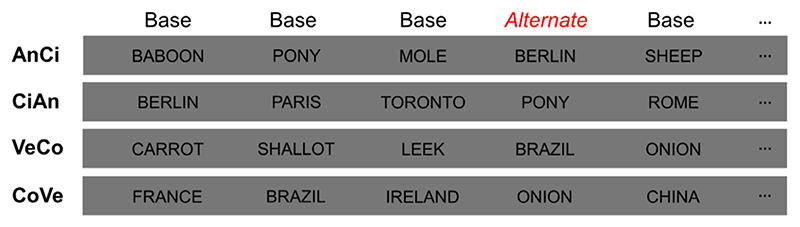
Experimental paradigm. Each trial lasted for 70 s, consisting in the presentation of 280 stimuli, including 70 alternate stimuli. Each contrast was repeated three times. Stimuli were presented at 4 Hz, with the stimuli of the alternate category inserted every 4 stimuli (at 1 Hz). Stimuli were randomly selected within each category of words, with no immediate repetition. Note that the original paradigm was done with French words. AnCi = animals as base stimuli and cities as alternate stimuli. CiAn = cities as base and animals as alternate. VeCo = vegetables as base and countries as alternate. CoVe = countries as base and vegetables as alternate.

In the present paradigm, different visual words belonging to the same semantic category (e.g., different animals) are presented at a relatively fast rate (4 stimuli/second, i.e., 4 Hz) while brain activity of neurotypical adult participants is recorded using high-density scalp EEG. Importantly, participants are not involved in any explicit categorisation task. Every 4 stimuli, words belonging to a different semantic category (e.g., different cities) appear at 1 Hz (Fig. 1). Based on evidence from these types of FPVS paradigms (e.g., Rossion et al., 2020), the rationale of the present study is the following: if the words presented at 1 Hz are systematically treated distinctly from the words presented at the base frequency (4 Hz), then they should evoke a common *selective* (i.e., differential) neural response, i.e., a marker of conceptual categorisation, at their exact frequency of stimulation (1 Hz).

## 2 Material and methods

### 2.1 Participants

Fifteen right-handed French-speaking volunteers were recruited from a pool of participants at the University of Louvain (Belgium). One participant’s data were excluded from the analyses because of neurological history, leading to a final sample of 14 participants (8 females, mean age = 23.28, SD = 2.29). The sample size was based on previous FPVS of word and face categorisation in human adults (e.g., Lochy et al., 2015, *N* = 10; Rossion et al., 2015, *N* = 12, respectively), considering that the FPVS approach is characterized by a very high signal-to-noise ratio (SNR) (Regan, 1989; Norcia et al., 2015). This high SNR is partly due to the large number of discrimination trials (i.e., here, word-semantic contrasts) recorded in a short amount of time for every individual tested (i.e., here, 70 discriminative responses for each of the 12 stimulation sequences, i.e., 840 trials in total). Participants all reported normal or corrected-to-normal vision. All participants signed a written consent form and received financial compensation at the end of testing. The experiment was approved by the Biomedical Ethical Committee of the University of Louvain (B403201111965). Participants were unaware of the goal of the study and the relative rates and ratios of the type of words presented in the study.

### 2.2 Stimuli

Four semantic categories (animals, cities, vegetables and countries) were selected. Each category contained 23 French words written in capital letters (font Verdana). Their length varied between 4 and 8 letters. Seen at 80 cm distance, they subtended about 5° to 11.8° (width) x 1.6° (height) of visual angle.

Two pairs of semantic categories were defined: animals/cities and vegetables/countries. Four contrasts resulted from this pairing: (1) names of animals as “base” stimuli and names of cities as “alternate” stimuli (AnCi), (2) cities as “base” and animals as “alternate” (CiAn), (3) names of vegetables as “base” and names of countries as “alternate” (VeCo), and (4) countries as “base” and vegetables as “alternate” (CoVe). Stimuli in each pair of categories were matched for their length (same number of 4-, 5-, 6-, 7- and 8-letters words in each category of the pair), and did not differ in terms of their bigram frequency (*t* = 0.209, *p* = 0.835 for the comparison of animals/cities; *t* = 1.397, *p* = 0.170 for the comparison of vegetables/countries) and number of orthographic neighbours (*t* = −0.687, *p* = 0.496 for the comparison of animals/cities; *t* = 0.404, *p* = 0.688 for the comparison of vegetables countries).

### 2.3 Experimental procedure

Participants were tested individually. They were seated at 80 cm from the computer screen while their brain activity was recorded at 512 Hz using a 128-channels BioSemi EEG system. Electrodes were referenced to the common mode sense and to the ground. Channel labels were converted to 10–20 system locations. Vertical and horizontal electrooculogram (EOG) was recorded by adding two electrodes at the outer canthi of the eyes and two electrodes above and below the participant’s right eye. Stimulation sequences were started manually when EEG signal was artefact-free. A fixation cross was first presented alone on a grey background, in the middle of the screen, for 2 to 5 s. Presentation of words gradually started in the middle of the screen with 2 s fade-in, then 70 s of stimulation, and ended with 2 s fade-out.

The experiment consisted in three repetitions of each contrast, resulting in a total of 12 stimulation sequences. Written words were presented at a frequency rate of 4 Hz, with each word being presented for 250 ms and replaced immediately by another word. A relatively slow frequency rate of 4 Hz (compared to stimulations with faces amongst objects at 6 Hz, e.g. Rossion et al., 2015, or words amongst pseudo-letters, e.g., Lochy et al., 2015), was selected based on several factors: the average silent reading rate for adults in English (i.e., about 4 words by second; Brysbaert, 2019), pilot data of the present experiment with several frequency rates and, finally, considering that 250 ms of stimulus duration should be largely sufficient for visual word recognition in the human brain (e.g., Maurer and McCandliss, 2008; Hauk et al., 2012). Alternate stimuli appeared periodically at a frequency rate of 1 Hz (1/4) within the rapid stream of base stimuli, ensuring sufficient spacing between alternate stimuli (i.e., 1 second) to capture full semantic categorisation responses (Fig. 1) (Hauk, 2016). Stimuli were selected randomly within each category, with no immediate repetition.

Participants were asked to focus on the fixation cross superimposed to words and press the keyboard space bar when the fixation cross changed its colour. Their task was thus unrelated to the process of interest and was used only to keep their attention focused on the screen. Colour changes randomly occurred 8 times for 500 ms within each trial. Accuracy and response times (RT) were automatically computed by the stimulation software running on Java. Accuracy was almost at ceiling in each contrast (accuracy range = 98.96-100%), and no significant difference was found in RT between the 4 contrasts (VeCo= 443 ms; CoVe= 456 ms; AnCi= 452 ms; CiAn= 444 ms). This indicates that the subjects’ attention was maintained constantly across the experiment.

### 2.4 Preprocessing of EEG data

EEG data were analysed using Matlab 2014 (The Mathworks) and the free software Letswave 5 (https://github.com/NOCIONS/Letswave5), following procedures validated in many FPVS-EEG studies (e.g., Rossion et al., 2020) but also detailed here. After importation of raw data files, continuous blocks of recording were aligned to the first block to correct for vertical jumps due to voltage drift during pauses. A bandpass filter was applied on EEG data at 0.1 and 100 Hz, followed by a notch filter at 50 Hz (0.5 Hz width). A downsampling to 256 Hz was computed to reduce file size and data processing time. Data files were then segmented on the basis of four stimulation start triggers (each trigger corresponding to one of the four contrasts), including two seconds before each trial. To get an overview of electrophysiological responses independently of the four types of stimulations, we merged the data from these contrasts at the segmentation phase.

Prior to channel interpolation, a single component accounting for blink artefacts detected on the electrode located below the right eye was removed for all participants, based on an independent component analysis (ICA) applied on the EEG data. Residual noisy electrodes (less than 5% per participant) were linearly interpolated. All electrodes were then re-referenced to the common average. Data files were segmented again using an integer number of 1 Hz cycles (= full cycles of one alternate + three base stimuli, to remove incomplete cycle at the end of the sequence), corresponding to 17,666 bins in total, which excluded fade-in and fade-out portions of the trial. Resulting epochs were averaged for each participant separately before transforming data to the frequency domain in order to increase signal-to-noise ratio (SNR).

### 2.5 Frequency-domain analysis of EEG data

A Fast Fourier Transform (FFT) was applied on these averaged epochs to perform frequency-domain analyses, and amplitude spectra were extracted for each individual. Three more transforms were computed on data files to (1) quantify the electrophysiological response in microvolts (baseline subtraction), (2) estimate the SNR (baseline division), and (3) determine whether significant responses were present at the conceptual categorisation and base frequencies (z-scores computation) (Retter and Rossion, 2016). The baseline subtraction was defined as the difference between the amplitude of the frequency bin of interest and the average amplitude of the 20 surrounding bins (10 on each side), excluding the immediately adjacent bin in case of remaining spectral leakage, and the local maximum and minimum amplitude bins to avoid projecting the signal in the neighbouring bins containing noise. SNR was computed in a similar way except that the amplitude of the bin of interest was divided by the average amplitude of the 20 surrounding bins (10 on each side). Z-scores were calculated by computing the difference between the amplitude of the bin of interest and the mean amplitude of the 22 surrounding bins (11 bins on either side, no exclusion of maximal or minimal bins) and dividing this value by the standard deviation of amplitudes in the 22 corresponding surrounding bins. FFT data were averaged across all 14 participants to compute topography maps and visualise the global response on the frequency spectrum. As for individual data, baseline-subtraction, SNR estimation and z-scores were computed on these grand-averaged data files.

To quantify the response at the frequency of category change, the sum of harmonics of the frequency of conceptual change was computed by segmenting individual FFT epochs into successive chunks centred on the bin containing the conceptual categorisation frequency (1 Hz, 2 Hz, 3 Hz, etc.) (Retter and Rossion, 2016). Each chunk contained 25 bins (i.e., a chunk length of 0.362 Hz), with the bin in the middle (the 13th bin, with 12 bins on each side) corresponding to the conceptual categorisation frequency. Based on the grand-averaged significant responses across electrodes (*z* > 1.65) for all contrasts averaged together, we extracted 7 chunks, corresponding to the first six harmonics of the conceptual categorisation frequency (i.e., 1, 2, 3, 5, 6 and 7 Hz), removing the 4th chunk corresponding to the base frequency (4 Hz). Resulting epochs were then summed. Individual results and grand-averaged data for each electrode across participants were examined. Baseline-subtraction, SNR estimation and z-scores were also calculated on the sum of harmonics. All amplitude values reported in the results section are baseline-corrected amplitudes.

The sum of base rate harmonics was computed following the same principle and chunking parameters than the sum of harmonics of the conceptual categorisation frequency (25 bins). Considering the greater amplitude of base responses, reflecting general visual responses synchronized to the frequency of presentation, the statistical threshold used to determine their significance (*z* > 3.1) was more conservative than the threshold used to detect significant conceptual categorisation responses. Based on the grand-averaged significant responses across electrodes for all contrasts together, we selected the sixteenth first base harmonics (from 4 to 64 Hz). These epochs were then summed. As for previous data files, individual and grand-averaged results were considered and transformed by the same manipulations as seen above (baseline sub-traction, baseline division and z-scores).

### 2.6 Statistical analysis

Significant electrodes were defined as described above, i.e., by calculating the difference between the amplitude at the bin of interest and the mean amplitude in the 22 surrounding bins (11 bins on either side) and dividing this value by the standard deviation of amplitudes in the 22 corresponding surrounding bins. False Discovery Rate (FDR) corrections were then applied to control for multiple comparisons (Benjamini and Hochberg, 1995).

Comparisons between contrasts were conducted using the SPSS software (IBM SPSS Statistics, Version 20.0). Paired t-tests and repeated-measures ANOVAs were used to investigate the effects of contrasts and regions-of-interest (ROI). A p-value under 0.05 was considered as statistical threshold for significance.

## 3 Results

### 3.1 General visual word stimulation

**Fig. 2:**
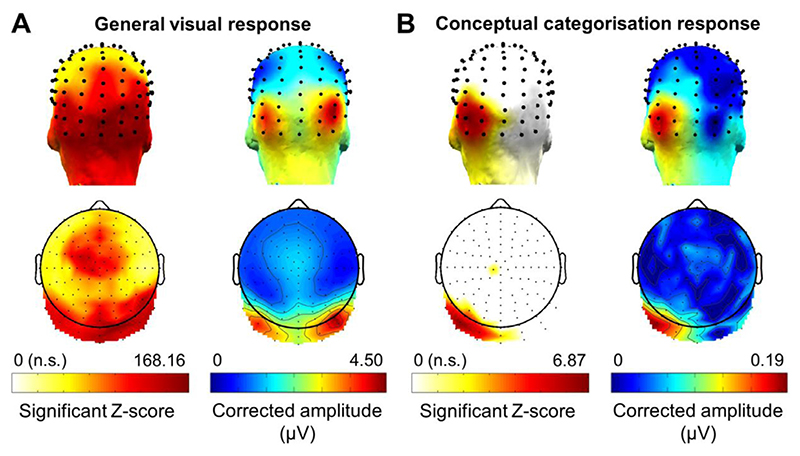
Topographical distribution of the electrophysiological responses at the general visual (A) and conceptual categorisation (B) frequencies (grand-averaged data, *n* = 14 participants). On the left of each panel are displayed 3D and 2D topographical maps of the distribution of FDR-corrected significant z-scores at the sum of harmonics (16 harmonics for the general visual frequency; 6 for the conceptual categorisation frequency). The white colour corresponds to non-significant responses at the frequency of interest. On the right of each panel, 3D and 2D topographical maps of baseline-corrected amplitudes (in microvolts) are showed at the sum of harmonics. Maximal amplitudes are located in the bilateral occipito-temporal regions for the general visual stimulation frequency, but are strongly left-lateralized at the conceptual categorisation frequency.

A strong neural response was expected at 4 Hz, the stimulation frequency reflecting general visual processes, and its harmonics (8 Hz, 12 Hz, etc.). When considering the FFT spectrum across contrasts, participants and electrodes, we found clear significant general visual responses (*z* > 3.1) up to the 16th harmonic (64 Hz). The sum of the 16 harmonics of the general visual frequency had an overall amplitude of 1.41 μV (± 0.85) and was associated with a very high SNR (*M* = 5.04 ± 1.90, across all electrodes). While all electrodes showed a significant response at the sum of base harmonics, even after FDR correction (z-scores’ range = 24.85–168.16), the largest amplitudes were found over bilateral occipito-temporal regions (maximal baseline-corrected amplitude at PO8: 4.50 μV) (Fig. 2A).

### 3.2 Semantic/conceptual categorisation

We first computed the response across contrasts over the sum of 6 harmonics of the conceptual categorisation frequency. While the z-score calculated on the grand-averaged of participants and electrodes revealed a significant response at the sum of harmonics (*z* = 2.4; *p* < 0.01), the maximal response appeared to be very focal, localized mainly over the left occipito-temporal cortex (Fig. 2B). Eleven electrodes (i.e., 8.6%) remained significant after FDR correction, with the large majority (10/11) being contiguous over the left occipito-temporal region: P7, P9, PPO5, PO7, PO9, PO11, O1, POI1, I1 and Oiz (z-scores’ range = 2.97–6.87; corrected p-values ranging from 0.02 to *p* < 0.0001). Amongst these electrodes, PO9 showed the largest response amplitude. Electrode C1h also showed a significant response at the sum of harmonics (*z* = 2.65, corrected p-value = 0.04) (Fig. 2B), but was not considered for further analysis because of its isolation from other significant electrodes. These observations reveal a clear periodic response at the frequency of conceptual category change, with a distinct topographical signature compared to the base rate response (Fig. 2).

**Fig. 3:**
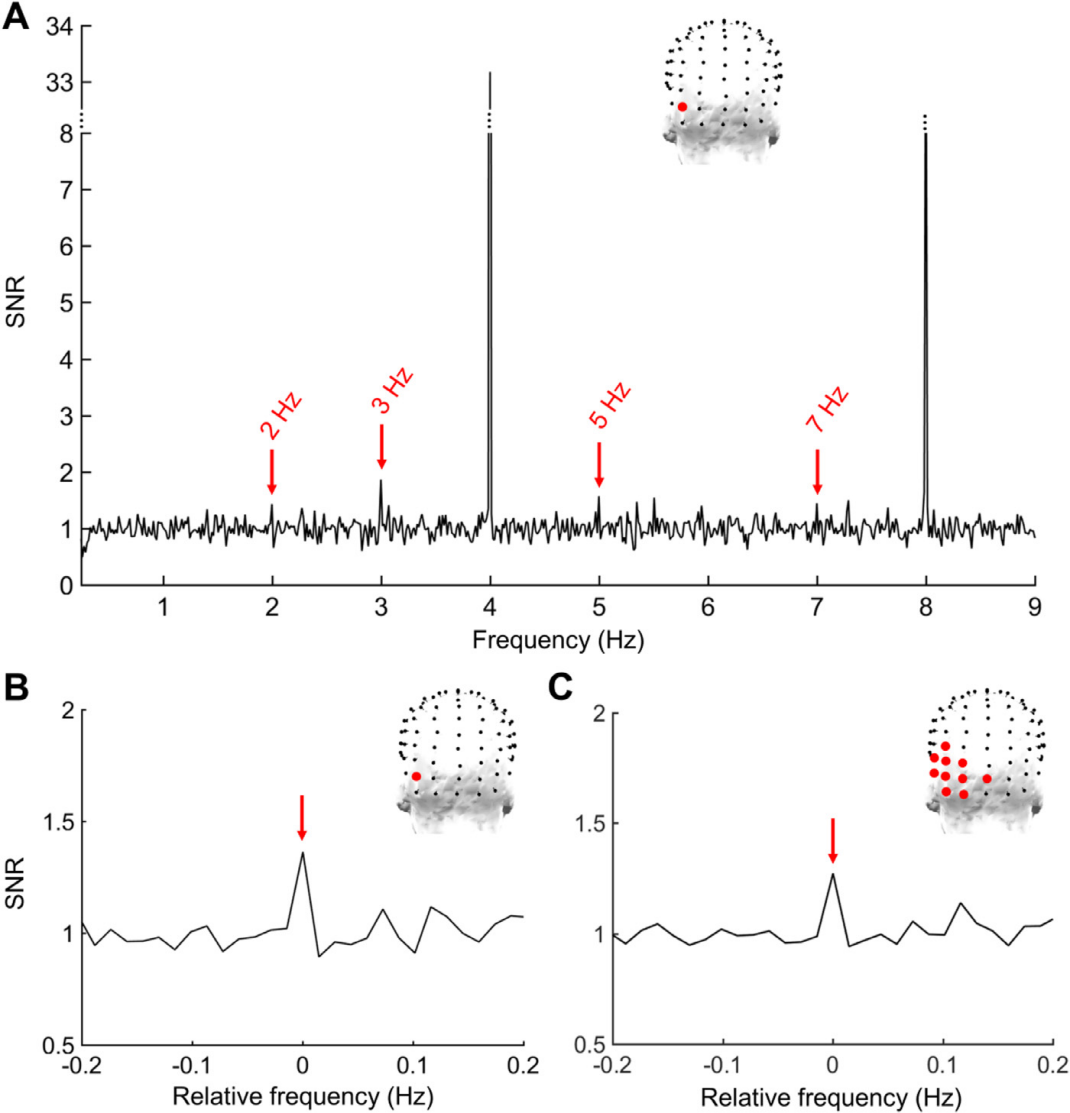
FFT spectrum of EEG responses over the left occipito-temporal region. A. Signal-to-noise ratio (SNR) EEG spectrum shown for electrode PO9 exhibiting the largest amplitude at the sum of harmonics (grand-averaged data, *n* = 14 participants). The location of this electrode is indicated with a red dot on the topographical head displayed above the EEG spectrum. The red arrows highlight the 4 harmonics for which a significant response (*z* > 1.65) is observed across subjects. Note that the harmonics at 1 Hz and 6 Hz for the conceptual categorisation frequency are not significant. B. SNR for the sum of 6 conceptual categorisation harmonics on electrode PO9 (a SNR of about 1.35 corresponding to 35% increase of signal relative to noise). The spectrum has been centred on the frequency bin of interest, with 0 corresponding to the sum of harmonics until 7 Hz, excluding the base harmonic at 4 Hz. C. SNR at the sum of 6 conceptual categorisation harmonics on the average of the 10 left-lateralised electrodes on which a significant (FDR-corrected) response was observed. The location of these electrodes is displayed on the topographical head above the EEG spectrum.

Fig. 3 shows the SNR of the response at the maximal electrode PO9 on the FFT spectrum (Fig. 3A and B), revealing clear responses at multiple harmonics up to 7 Hz, as well as the sum of harmonics at the average of these 10 FDR-corrected significant occipito-temporal electrodes (Fig. 3C). Even though only the left occipito-temporal region was associated with a significant conceptual categorisation response, this response might not be significantly different from the homologous region of the right hemisphere, considering the important role that the right temporal cortex also plays in semantic processing (Lambon Ralph et al., 2017 for a review). To test this, we grouped the significant left OT electrodes (P7, P9, PPO5, PO7, PO9, PO11, POI1, O1, and I1) into a data-driven left ROI and defined a right counterpart ROI with the corresponding electrodes (P8, P10, PPO6, PO8, PO10, PO12, POI2, O2 and I2). This analysis excluded significant electrode Oiz located on the midline. A paired *t*-test on the average amplitude in these two ROIs showed a significantly greater amplitude of the response in the left (*M* = 0.14 μV ± 0.12) than right ROI (*M* = 0.04 μV ± 0.07; t(13) = 3.634, *p* = 0.003).

For the sake of completeness, we also ran an unbiased analysis by comparing the left and right occipito-temporal ROIs that were defined a priori from a ROI definition on our 128 channel EEG system (see Rossion et al., 2020). Each ROI included 9 lateralized occipito-temporal electrodes (i.e., CPP5h, P5, P7, P9, PPO3, PPO5, PO7, PO9 and PO11 vs. CPP6h, P6, P8, P10, PPO4, PPO6, PO8, PO10 and PO12). A paired *t*-test on the average amplitude in these ROIs showed again that EEG amplitudes for the conceptual categorisation response were significantly larger over the left (*M* = 0.11 μV ± 0.10) than the right (*M* = 0.03 μV ± 0.06) occipito-temporal region (t(13) = 2.576, *p* = 0.023).

### 3.3 Conceptual categorisation of living vs. non-living items

Since the contrasts used in the study could be separated into two groups, i.e., animals and vegetables as referring to common names of living entities; cities and countries to proper names of non-living entities, we performed a further analysis, separately averaging the data for contrasts AnCi and VeCo together and the contrasts CiAn and CoVe together. The average of contrasts in each group (living or non-living contrasts) was done before FFT to increase SNR.

For the base rate response, there was no significant difference in the mean amplitude on the whole scalp across the two types of contrasts (t(13) = 1.984, *p* = 0.069), with highly similar bilateral occipito-temporal topographies. All electrodes showed a significant FDR-corrected response at the sum of base harmonics (living contrasts: z-scores’ range = 17.90-125.98; non-living contrasts: z-scores’ range = 15.48-158.11). The largest amplitudes were again found over bilateral occipito-temporal regions, with a maximal baseline-corrected amplitude at electrode PO8 in both living (max. 4.44 μV) and non-living (4.30 μV) contrasts.

**Fig. 4:**
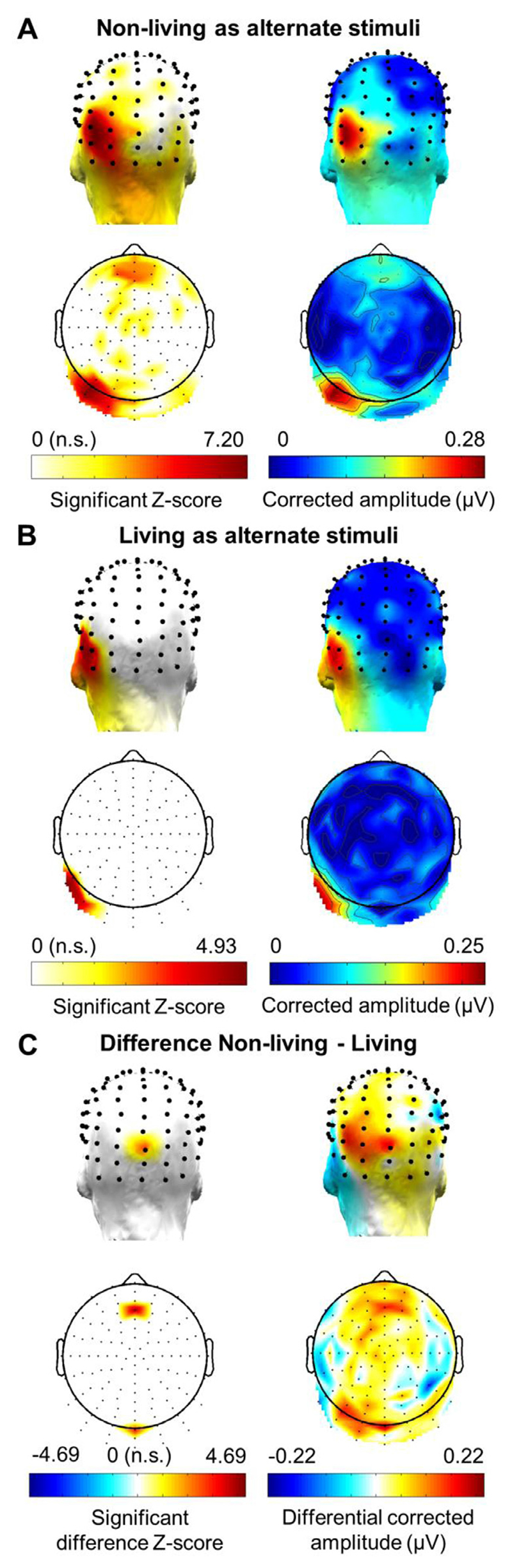
Topographical distribution of the electrophysiological responses at the conceptual categorisation frequencies according to the type of contrast (grand-averaged data, *n* = 14 participants). A. Topographical distribution of the response when alternate stimuli are non-living items. White-to-red 2D and 3D topographies display the distribution of FDR-corrected significant z-scores at the sum of 6 harmonics, with the white colour corresponding to non-significant responses at the conceptual categorisation frequency. Blue-to-red 2D and 3D topographies show the distribution of baseline-corrected amplitudes (in microvolts) at the sum of harmonics. B. Topographical distribution of the response when alternate stimuli correspond to living items. C. Difference map showing the distribution of response when subtracting EEG responses at the sum of harmonics in the living contrasts from EEG responses at the sum of harmonics in the non-living contrasts. While only two electrodes show a significant FDR-corrected response, the amplitude map indicates more dorsal and medial occipito-temporal responses in the non-living contrasts and more ventro-lateral occipito-temporal responses in the living contrasts.

Most interestingly, for the conceptual categorisation response, the maximal baseline-corrected amplitude was found at PO7 (max. 0.28 μV) for non-living items and at P9 for living items (max. 0.25 μV), but there was no significant difference in the mean amplitude across the whole scalp between the two contrasts (t(13) = −1.370, *p* = 0.194). A large number of electrodes (*n* = 41) showed a significant FDR-corrected response at the sum of harmonics in the non-living contrasts, and were mostly located over the left dorsal occipito-temporal region and over the fronto-central region (Fig. 4A). On the contrary, only 4 electrodes remained significant in the living contrasts after FDR correction, forming a small cluster in the left lateral and more ventral occipito-temporal region (electrodes P7, P9, PO9 and PO11; see Fig. 4B). To assess whether there was a significant difference between the two types of contrasts over the whole scalp, we subtracted the FFT spectrum of the response to living contrasts as alternate stimuli from the FFT spectrum of the response to non-living contrasts as alternate stimuli, and calculated z-scores and baseline-corrected amplitudes on this differential FFT spectrum (Fig. 4C). Z-scores were then FDR-corrected. Although this sub-traction showed that only two electrodes (Oz and AFFz) remained significant after correction for multiple comparisons, visual inspection of the differential response over the left occipito-temporal region indicated larger amplitudes over dorso-medial electrodes in the non-living contrasts (positive amplitudes) and larger amplitudes over ventro-lateral electrodes in the living contrasts (negative amplitudes).

Considering these notable differences, we tested whether maximal electrodes were significantly different in one contrast compared to the other. To do so, we performed a two-way repeated-measures ANOVA with the factors *Contrasts* (2: living and non-living) and *Electrodes* (10: significant occipito-temporal electrodes as found in the main analysis based on the average of all contrasts; see Fig. 3C). There was no effect of *Contrasts* (F(1,13) = 0.328, *p* = 0.576) but a main effect of *Electrodes* (F(9,117) = 2.920, *p* = 0.045). Most importantly the significant interaction effect between *Contrasts* and *Electrodes* (F(9,117) = 3.403, *p* = 0.001) indicates that the distribution of activity on the scalp for the conceptual categorisation response significantly differs between the two contrasts, in line with the observed dorsal/medial (non-living) vs. ventral/lateral (living) dissociation (Fig. 4).

## 4 Discussion

The goal of this study was to demonstrate and characterize automatic semantic/conceptual categorisation using a novel application of FPVS-EEG and written word stimuli. We show here that automatic conceptual categorisation processes can be captured through brief conceptual category changes using written words without requiring any explicit task. Clear electrophysiological responses were observed over the left occipito-temporal region at the exact frequency of category change (i.e., 1 Hz) and its specific harmonics, thus opening the way for using and further developing this approach as an implicit marker of semantic memory abilities. While generic visual responses to words occurring at the 4 Hz rate were bilateral over the occipito-temporal cortex, responses reflecting conceptual categorisation processes were strongly left-lateralised over this cortical region. Interestingly, we also found relatively more medial and dorsal occipito-temporal responses for categorisation of non-living items while responses were more lateral and ventral for categorisation of living items.

### 4.1 Left lateralisation of written word-based conceptual categorisation responses

The neural periodic response at the frequency of word category change was found over the left occipito-temporal region. This pattern of response is consistent with previous studies showing a strong involvement of the left temporal lobe in the representation of conceptual knowledge for written words. Several functional neuroimaging studies have shown a left-lateralised activation of the ventral temporal lobe during semantic tasks with written words (semantic matching, semantic judgement, naming; Marinkovic et al., 2003; Seghier and Price, 2011; Rice et al., 2015; Binney et al., 2016; Hoffman and Lambon Ralph, 2018). Furthermore, numerous patient studies have shown an association between left (mainly anterior) temporal lesions and lower performance on similar written word-related semantic tasks (e.g., word version of the Pyramids and Palm Trees test or Camel and Cactus test), reinforcing the view of a greater involvement of left temporal structures in written word conceptual processing (Acres et al., 2009; Butler et al., 2009; Snowden et al., 2012, 2018; Rice et al., 2018a, 2018b). It should be noted that, unlike the majority of previous studies, our study was not based on any explicit tasks, suggesting that left temporal structures were automatically recruited in relation with the periodic category change.

Although most of these studies have related verbal (written word) semantic processing with the anterior temporal lobes, the maximal amplitudes of the semantic categorisation responses were located on relatively posterior, occipito-temporal regions in our study. While this result can be surprising, no recording channel was positioned over the basal temporal area which is the closest from deep anterior temporal structures. This lack of basal anterior electrodes in our EEG study might be responsible for the rather posterior location of the locus of maximal responses, potentially occulting another more anterior source of activity. In fact, a foundational MEG study showed that processing visual (written word) semantic information was associated with an early involvement of the left ventral occipito-temporal area, similarly to what was found in our study, followed by a later recruitment of anterior temporal regions (Marinkovic et al., 2003). This view of progressive integration of semantic information from modality-specific regions to transmodal anterior temporal structures has been more recently validated by several studies in fMRI (Hoffman and Lambon Ralph, 2018; Jackson et al., 2018; Bajada et al., 2019; see also Lambon Ralph et al., 2017), suggesting that the response observed in scalp EEG here might reflect only the posterior part of the neural sources recruited during conceptual categorisation. Future studies allowing more precise recordings from anterior temporal regions (i.e., intracranial EEG, or magnetoencephalography, MEG) will be needed to clarify this issue.

In comparison with the strong left lateralisation of conceptual categorisation responses, generic visual responses to words at 4 Hz were located bilaterally over occipito-temporal regions, with no hemispheric dominance. This is slightly surprising given the left hemispheric dominance in letter and word representation (Cohen et al., 2000; Wandell, 2011) including data obtained during FPVS (Lochy et al., 2015, 2018). However, this generic visual response encompasses many low-and high-level visual processes beyond word processing, including typically right-lateralised regions related to the processing of visual inputs.

### 4.2 Quantitative and qualitative differences depending on the semantic domain

It is generally assumed that semantic representations of living things are more tightly packed than those of non-living items, because stimuli within the former category are highly similar to each other, sharing many more common and intercorrelated features (e.g., has legs, can walk) than non-living stimuli (Gonnerman et al., 1997; Garrard et al., 2001). As a consequence, retrieving names or other semantic information about living things has often been shown to be more difficult than doing so with non-living items (Coppens and Frisinger, 2005; Lambon Ralph et al., 2007). The same principle applies to our study: more tightly packed concepts will form a more homogeneous base category, and thus provide a clearer semantic contrast to the alternate stimuli within the sequence (albeit the semantic difference between living and non-living concepts is considerable). While this might translate into larger differential responses when non-living items are periodically presented amongst a fast rate of living items compared to the reverse pattern, we found only a numerical advantage at this level (Fig. 4), with no significant difference between the two orders of contrasts. However, overall, the response to alternate non-living items was more distributed (i.e., 41 significant electrodes, mainly located over the left occipitotemporal region but also fronto-central region) than the focal response (i.e., cluster of 4 electrodes) to alternate living items.

Interestingly, we also found a graded difference in the relative topology of the response for living (ventral) vs. non-living (more dorsal) stimuli. This result might align with classical differences observed in ventral occipito-temporal regions (Hasson et al., 2002) in which animate items engage more medial, ventral areas and manmade items activate more lateral ventral occipito-temporal and posterior temporal areas. There were also more prominent frontal responses for non-living items (cities and countries), consistent with frontoparietal engagement for spatial coding (Cona and Scarpazza, 2019). These results fit with distributed models of semantic memory and high-order visual processing in which different sources of sensorimotor knowledge are bound together via interaction with a high-order anterior-temporal-lobe-centred multimodal hub (Chen et al., 2017). In posterior regions of the ventral occipito-temporal cortex, the differential engagement of the foveally-driven lingual gyrus-posterior fusiform for animate items, may reflect the greater visual processing demands for differentiation of the visually-overlapping nature of animate and subordinate exemplars (Hasson et al., 2002). As already stated above, recordings focusing on these structures will be needed to further understand the cerebral bases of the automatic semantic categorisation observed in this FPVS paradigm.

### 4.3 Relevance of a word-based FPVS paradigm

Considering its numerous advantages, using FPVS to study semantic/conceptual categorisation processes is extremely appealing. Up to now, the FPVS approach had been tested to probe automatic semantic categorisation using pictures of object categories (Stothart et al., 2017; Milton et al., 2020) or faces (i.e., comparison of natural images of unfamiliar faces with famous faces associated with rich semantic information; Zimmermann et al., 2019 Yan and Rossion, 2020). However, selective neural responses to pictures or photographs of familiar items can be partly due to differential physical features rather than semantic knowledge. Interestingly, none of these studies reported a strong (left) lateralization of the categorisation response as found here.

At a fundamental level, this study demonstrates that the FPVS-EEG approach can be used to study conceptual processes with written word stimuli, by showing for the first time that valid electrophysiological responses can be recorded in response to a periodic word-related conceptual category change. Admittedly, in this initial demonstration, we used only four semantic categories referring to concrete and well-contrasted entities (i.e., animals vs. cities, vegetables vs. countries). However, we expect the approach to reveal implicit measures of finer-grained (e.g., vegetables vs. animals, mammals vs. birds, etc.) and multi-level conceptual categorical distinctions in future studies, with variable amplitudes and scalp topographies. In this way, our paradigm opens the way for future studies with a full range of concepts, including abstract, non-picturable items, to better understand the neural basis of semantics more generally.

An important methodological aspect of the present FPVS-EEG approach of categorisation is the use of multiple variable exemplars for each contrasted category (Rossion et al., 2015), ensuring that the neural conceptual categorisation response is not due to specific low-level physical distinctions between highly repeated stimuli (e.g., if only 2 or 3 written names of animals and cities were used here for instance). This is the main reason why a relatively large number of words referring to different items (i.e., 23) rather than a limited set for the alternate category (e.g., 8) was used, matching the number of different items between the two contrasted categories. A potential caveat of this methodological choice is the unequal number of repetitions for the base and alternate stimuli presented during the course of the experiment (i.e., 3 times more repetitions for each base stimulus). However, the neural conceptual categorisation response is unlikely to be generated or even modulated by the unequal number of repetitions, for two reasons. First, providing that alternate stimuli do not appear too close to each other in time to avoid response overlap, previous studies of natural image categorisation have shown that the number of repetitions for base and alternate stimuli has no impact whatsoever on the amplitude of the categorisation response (Retter and Rossion, 2016, for presentation ratios of faces amongst objects of 1/5 to 1/11 with the same stimulus set). Second, with 23 items (pseudo-)randomly presented in the base category, one would expect putative repetition effects to increase over the course of the stimulation sequence and the experiment. This is clearly not the case in the present study, in which the conceptual categorisation response was stable across the three repeated stimulation sequences (see Supplementary Material, Figure S1A) and within sequences (divided into three 23-s epochs; Supplementary Material, Figure S1B).

Finally, at a clinical level, the present study lays the foundation for future electrophysiology-driven clinical tools to assess semantic abilities. Semantic impairments are frequent in the neurological population (Lambon Ralph et al., 2012; Vogel et al., 2014; Lehrner et al., 2017), but their evaluation is complicated by the explicit nature of neuropsychological tests, often requiring the involvement of executive and non-semantic language processes. With this technique, the only task to perform is to detect colour changes in the fixation cross presented in the middle of the screen, on top of the stimuli, thus not requiring the participant to produce any explicit output. This implicit task, in addition to the many advantages of FPVS (objectivity, sensitivity, short testing time), makes it a potentially powerful tool to provide an electrophysiological biomarker of semantic abilities in the clinical population, especially in the cases where language abilities are impaired following stroke, dementia or other neurological disorders.

## 5 Conclusions

In summary, our results reveal that automatic conceptual categorisation processes can be readily captured with a fast periodic visual approach using written words. Clear electrophysiological responses were found exactly at the frequency of conceptual change and its harmonics over the left occipito-temporal region. Our results also highlighted topographic differences depending on whether the alternate stimuli were living items, associated with relatively more ventral and lateral activity, or non-living items, associated with relatively more dorsal and medial activity. The present study lays the foundation for future studies to develop this approach as an implicit marker of semantic memory abilities with written language.

## Supplementary Material

Supplementary material associated with this article can be found, in the online version, at doi:10.1016/j.neuroimage.2021.118228.

Supplementary Material

## Data Availability

Electrophysiological data will be made available on a data repository upon acceptance of the manuscript.
