# Supplementary material for: Implicit, automatic semantic word categorisation in the left occipito-temporal cortex as revealed by fast periodic visual stimulation
Source: Neuroimage. Author manuscript; Available in PMC 2022 Jul 28. (PMC7613186; doi:10.1016/j.neuroimage.2021.118228)
Supplement: Supplementary Material [file EMS151055-supplement-Supplementary_Material.docx]

# Supplementary material


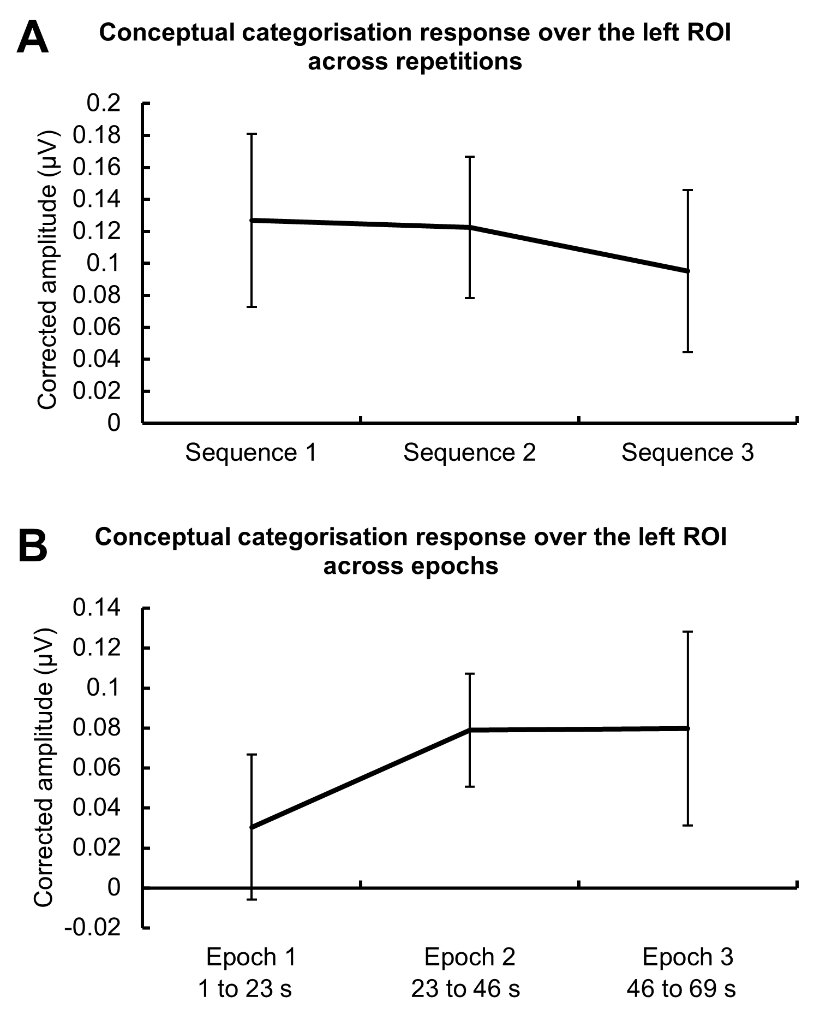


**Figure S1. Evolution of the conceptual categorisation response (average of all contrasts) across stimulation sequence repetitions (A) and within sequences (B) (grand-averaged data: n = 14 participants). A.** Baseline-corrected amplitudes (in microvolts) at the sum of 6 harmonics of the conceptual categorisation frequency, showed separately for each sequence number. Amplitudes are displayed for the average of the 10 significant occipito-temporal electrodes highlighted in the main analysis. Error bars represent standard error from the mean. **B.** Baseline-corrected amplitudes (in microvolts) at the sum of 6 harmonics of the conceptual categorisation frequency, showed separately for each 23-seconds epoch (average across repetitions). Amplitudes are displayed for the average of 10 significant occipito-temporal electrodes. Error bars represent standard error from the mean.

## Analysis by sequences

Methods. This analysis was carried out on datafiles obtained after the second segmentation (according to bin number). Rather than averaging all sequence repetitions after the second segmentation, each sequence was processed as an individual file. Frequency-domain analyses were then performed exactly as described in the main methods section.

Results. We computed one-way repeated-measures ANOVAs to compare the amplitudes across the three sequences across the 10 significant occipito-temporal electrodes highlighted in the main analysis (P7, P9, PPO5, PO7, PO9, PO11, O1, POI1, I1 and Oiz). There was no difference between sequences across the left occipito-temporal region (F(2,26)=0.110, p=0.896), providing no evidence for repetition effect across sequences (see **Supplementary Figure S1A**).

## Analysis by epochs

Methods. This analysis was carried out on datafiles before transforming them into the frequency domain (average of contrasts in the time domain). Datafiles of each sequence were first segmented into three epochs of equal length. Since the total length of a sequence was 70 seconds (after the removal of fade-in and fade-out parts), each epoch lasted 23 seconds. After this segmentation into three epochs, the first, second and third 23-s epochs of each sequence were averaged separately. Datafiles were then transformed into the frequency domain through FFT. Similarly to the main analysis, we computed the sum of harmonics of the frequency of conceptual change by segmenting individual FFT spectra into successive chunks centred on the bin containing the conceptual categorisation frequency. Each chunk contained 25 bins as in the main analysis, but the frequency resolution was lower because of smaller epoch length (23 seconds here vs. 70 seconds in the main analysis). Thus, a larger chunk length (i.e., 1.09Hz) was required to contain the same number of bins as in the main analysis. Apart from this small difference in the chunking parameters, all other analysis steps were the same as the main analysis.

Results. A one-way repeated-measures ANOVAs was performed to compare the amplitudes across the three 23-s epochs across the 10 significant occipito-temporal electrodes highlighted in the main analysis (P7, P9, PPO5, PO7, PO9, PO11, O1, POI1, I1 and Oiz).There was no difference between epochs across the left occipito-temporal region (F(2,26)=0.719, p=0.497), providing no evidence for repetition effect across epochs, i.e., within sequences (see **Supplementary Figure S1B**).
